# Zamzam Water in Cancer and Beyond: Preclinical Cellular Effects and Proposed Mechanistic Hypotheses

**DOI:** 10.3390/ijms27209308

**Published:** 2026-10-20

**Authors:** Md. Sanower Hossain, Anwar Parvez, Md. Tarakul Islam, Shakil Ahmmed, Md. Hasibul Hasan, Peni Indrayudha, Hamzeh Alkhatib, Md. Hanif Munshi, Hasna Banu, Erindyah Retno Wikantyasning, Iswandi, Ambarwati Ambarwati

**Affiliations:** 1 Faculty of Pharmacy, Universitas Muhammadiyah Surakarta, Sukoharjo 57162, Indonesia; peni.indrayudha@ums.ac.id (P.I.); erindyah.rw@ums.ac.id (E.R.W.); 2 Faculty of Pharmacy and Health Sciences, Royal College of Medicine Perak, Universiti Kuala Lumpur, Ipoh 30450, Malaysia; hamzeh.alkhatib@unikl.edu.my; 3 Department of Pharmacy, Faculty of Health and Life Sciences, Daffodil International University, Dhaka 1216, Bangladesh; 4 Department of Food Engineering, Gopalganj Science and Technology University, Gopalganj 8105, Bangladesh; 5 Department of Biochemistry and Molecular Biology, Bangladesh Agricultural University, Mymensingh 2202, Bangladesh; 6 Department of Applied Chemistry and Chemical Engineering, Gopalganj Science and Technology University, Gopalganj 8105, Bangladesh; 7 Department of Pharmacy, Gopalganj Science and Technology University, Gopalganj 8105, Bangladesh; 8 Faculty of Pharmacy, Universitas Setia Budi, Surakarta 57127, Indonesia; iswandi@setiabudi.ac.id; 9 Department of Biological Education, Faculty of Teacher Training and Education, Universitas Muhammadiyah Surakarta, Sukoharjo 57162, Indonesia; amb184@ums.ac.id

**Keywords:** Zamzam water, cancer biology, molecular mechanisms, physicochemical properties, redox signaling, apoptosis, mitochondrial function, complementary oncology

## Abstract

Zamzam water (ZW), a naturally occurring alkaline mineral water with a distinctive ionic and trace-element composition, has attracted growing scientific interest due to its reported pharmacological properties and emerging biological effects relevant to cancer research. This review provides a comprehensive mechanistic synthesis that links the physicochemical characteristics of ZW to molecular and cellular processes implicated in cancer biology. Integrating evidence from cancer biology, redox pharmacology, mitochondrial physiology, and cellular signaling, this review examines how the unique mineral composition and mildly alkaline pH of ZW may collectively influence pathways associated with the hallmarks of cancer. Preclinical studies demonstrate context-dependent modulation of cell proliferation, apoptosis, oxidative stress, mitochondrial function, cell-cycle progression, and signaling pathways, including mitogen-activated protein kinase (MAPK) and cyclic AMP (cAMP) networks, across multiple cancer models. Beyond its reported antiproliferative activity, ZW also exhibits antioxidant, anti-inflammatory, and organ-protective effects in non-malignant systems. Importantly, this review proposes a conceptual framework for investigating the relative contributions of mineral ions, ionic homeostasis, redox regulation, and physicochemical properties to the biological effects of ZW, and identifies key mechanistic and methodological gaps. ZW has exhibited cancer-associated biological effects in experimental animals; however, its therapeutic effectiveness has yet to be validated. Future studies integrating standardized physicochemical characterization, molecular validation, multi-omics approaches, and clinical investigations are required to establish its translational relevance in complementary oncology.

## 1. Introduction

Cancer represents a profoundly heterogeneous group of diseases characterized by uncontrolled cellular proliferation, invasion, and metastasis, arising from intricate interactions among genetic, epigenetic, metabolic, and environmental factors. Meanwhile, patients’ psychological and psychosocial states may further influence disease progression and therapeutic outcomes [1,2]. Cancer thus remains the second leading cause of death globally after cardiovascular disease, accounting for nearly 271 million disability-adjusted life-years (DALYs) (255–285 million) in 2023. Strikingly, 57.9% of new cases and 65.8% of cancer deaths occurred in low- to upper-middle-income countries, where limited access to early detection and treatment continues to exacerbate mortality rates. Projections indicate a further escalation to 30.5 million incident cases and 18.6 million deaths by 2050—a 60–75% increase from 2024—driven primarily by demographic transitions, population aging, and persistent exposure to modifiable risk factors [3].

Reactive oxygen species (ROS) play a paradoxical role in these processes: while physiological levels of ROS are essential for intracellular signaling, excessive accumulation induces oxidative stress, DNA damage, and oncogenic transformation [4,5]. Conversely, the deliberate elevation of ROS in tumor cells has been proposed as a therapeutic strategy to trigger apoptosis and suppress tumor growth [6,7]. Conventional cancer therapies such as chemotherapy and radiotherapy exploit this mechanism, but their clinical success remains constrained by systemic toxicity and the emergence of resistance. These challenges have stimulated growing interest in natural or mineral-based adjunctive therapies that can modulate oxidative and metabolic pathways while minimizing collateral tissue damage [8,9].

Alkaline and mineral-enriched waters have recently emerged as a novel research focus in cancer biology. Electrolyzed-reduced water (ERW), characterized by its high pH, low oxidation potential, and reduced dissolved oxygen, has demonstrated antiproliferative and pro-apoptotic effects in multiple cancer cell lines [10,11,12,13]. Zamzam water (ZW), sourced from the historic Zamzam well in Mecca, Saudi Arabia, is a naturally alkaline, mineralized water with a unique ionic profile that distinguishes it from ordinary potable water. Comprehensive chemical analyses have identified sodium, potassium, calcium, magnesium, selenium, lithium, and trace arsenic as its predominant components [14,15,16].

ZW’s alkaline pH (7.9–8.0) and mineral content have been associated with antioxidant, anti-inflammatory, and antimicrobial properties in several experimental models [17,18,19,20]. Given that many of these minerals, particularly selenium, lithium, and arsenic, are known modulators of apoptosis, oxidative stress, and cellular signaling, it is biologically plausible that ZW may exert measurable anticancer effects. For instance, selenium compounds have been reported to inhibit angiogenesis and promote apoptosis [21,22]; lithium modulates Wnt/β-catenin and GSK-3β pathways [23]; and arsenic trioxide (As_2_O_3_) has demonstrated potent cytotoxicity against leukemia and solid tumor cells [24,25,26]. Furthermore, alkalinity itself may modulate the tumor microenvironment by inhibiting invasion and metastasis through extracellular pH correction [27,28,29,30].

Notwithstanding the growing interest, the biological importance of ZW is still not fully comprehended. The majority of existing research relies on in vitro experimental frameworks. At the same time, scholarly data on pharmacologically significant exposure levels, active constituents, molecular targets, long-term safety, and in vivo efficacy remain scarce. This study provides an in-depth assessment of the current understanding of the physicochemical characteristics, mineral constituents, and documented pharmacological effects of ZW, with a focus on its potential significance in cancer research. This manuscript covers possible cellular mechanisms, existing experimental evidence, limitations, and future research avenues necessary to elucidate the biological and translational significance of ZW.

## 2. Chemical Composition and Physicochemical Properties of Zamzam Water

The unique hydrochemical profile of ZW underpins much of its proposed biological and pharmacological activity. ZW is characterized by a high mineralization index, moderately alkaline pH, and stable ionic balance, distinguishing it from both typical groundwater and commercially bottled mineral waters. Analytical investigations employing techniques, such as inductively coupled plasma (ICP), neutron activation analysis, and ionic chromatography, have consistently demonstrated that ZW contains an elevated concentration of essential electrolytes and trace elements, notably sodium (Na^+^), calcium (Ca^2+^), magnesium (Mg^2+^), chloride (Cl^−^), and sulfate (SO_4_^2−^), alongside smaller amounts of selenium (Se), lithium (Li), zinc (Zn), and arsenic (As) [14,31].

Comparative studies indicate that ZW has a Total Dissolved Solids (TDS) concentration of 3000–3500 mg/L, placing it in the upper mineralization range for potable waters. Its pH typically ranges from 7.9 to 8.0, conferring mild alkalinity, which has been proposed as a contributing factor to its reported antioxidant and potential anticancer properties [31,32]. The water’s low oxidation–reduction potential and stable ionic ratios confer resistance to microbial contamination and chemical degradation, accounting for its long-standing physical purity despite environmental exposure [15].

Despite the mineral composition of ZW suggesting a possible biochemical framework for exploring its biological effects, the presence of certain minerals should not be interpreted as confirmation of therapeutic benefits (Table 1). The chemical structure, concentration, bioavailability, exposure duration, and interactions among numerous components in the aqueous solution all affect the body’s response to minerals. Thus, rather than confirming the factors influencing the effects of ZW, correlations between specific minerals and pharmacological pathways suggest potential mechanisms. Table 1 reports the absolute concentrations of individual mineral constituents in ZW; however, none of the compositional analyses reported include inter-elemental ratios (e.g., Na^+^:K^+^, Ca^2+^:Mg^2+^) that could further influence physiological or pharmacological responses. Because ionic ratios, rather than isolated absolute concentrations, are known to affect membrane transport, electrophysiological stability, and enzymatic cofactor competition, omitting ratio-based characterization highlights a considerable shortcoming in current compositional research on ZW. The mineral composition of ZW, while contributing to its bioactivity, also demands careful toxicological evaluation. Most trace and heavy metals, including lead (Pb), cadmium (Cd), chromium (Cr), and manganese (Mn), are found at concentrations well below the World Health Organization (WHO) permissible limits for drinking water [33]. As levels in ZW have been documented variably in different studies, with some source-collected samples showing concentrations beneath the detection limit [15], while others report values within acceptable ranges (0.006–7.728 µg/L) [34]. Certain samples, which were transported and stored overseas before testing, exhibited levels approximately three times the WHO limit of 10 µg/L (averaging ~27 µg/L) [16]. This discrepancy has been attributed to variations in sample collection sites, transportation methods, storage times, and analytical procedures, and it remains an unresolved issue in the compositional literature on ZW. Thus, any assertion of a single fixed As value for ZW should be approached with skepticism. This trend indicates that methods of collection, transportation, and storage are better-documented sources of variability than seasonal variations, which have not been systematically examined for ZW. Seasonal fluctuations in As levels are well documented in other groundwater systems, and a comparable mechanism is plausible given ZW’s alluvial aquifer origin; nonetheless, this assertion is insufficiently supported and should be regarded as a hypothesis. Standardized sampling that is controlled for place and time, preferably with repeated seasonal collections at identical sites, is essential to differentiate genuine compositional variability from processing artifacts. Se concentrations (approximately 2.5 µg/L) fall comfortably within safety margins, contributing antioxidant potential without toxicological risk. Slightly higher readings of uranium (U ≈ 1.6 Bq/L) and thorium (Th ≈ 1.2 Bq/L) have been reported compared with WHO’s radiological guideline of 0.1 Bq/L; however, these values are still several orders of magnitude below the threshold associated with human health hazards [31].

From a hydrogeochemical perspective, ZW is classified as a sodium-chloride (NaCl)-type water of meteoritic origin, formed through prolonged rock–water interactions within the alluvial aquifer beneath the Mecca Basin [31,35]. Isotopic analysis indicates minimal anthropogenic influence, underscoring the water’s geochemical stability. Trace levels of naturally occurring radionuclides, including 40K, 137Cs, 226Ra, and 232Th, fall within the global background radiation range, thereby posing negligible radiological risk. It is crucial to understand that the compositional analysis of ZW thus far has predominantly focused on inorganic ions and trace elements, employing methods such as ICP, ion chromatography, and neutron activation analysis. Current evidence suggests that ZW has a minimal total organic carbon concentration [16]. However, this overall assessment does not preclude the presence of low-abundance organic components, including enzymes, amino acids, peptides, or other bioactive cofactors, which may evade detection by standard bulk organic carbon analysis. No impartial metabolomic or proteomic analysis of ZW has been released thus far. As a result, a non-mineral influence on the biological effects discussed in this review cannot be dismissed given the existing evidence, and forthcoming multi-omics analyses (Section 8) should specifically incorporate untargeted metabolomic and proteomic assessments to address this deficiency.

**Table 1:** Mineral composition of Zamzam water and its potential biological relevance: interpreting current evidence and limitations.

Minerals	Amount (mg/L)	Reported Range (mg/L)	Biological Relevance	Reported Biological Relevance of the Element/Ion	Evidence Specifically Related to ZW	Current Interpretation and Limitations	References
Sodium (Na^+^)	55	10–100	Osmotic balance	Na contributes to extracellular osmotic balance, regulation of the membrane potential, and cellular homeostasis. Alterations in Na balance can influence cellular signaling processes.	The alkaline and mineral-laden properties of ZW have been linked to biological effects in experimental research; however, direct anticancer effects, particularly those attributed to Na, have yet to be validated.	Na is a vital electrolyte; nevertheless, its levels in ZW are unlikely to elicit pharmacological responses. The role of salt in anticancer efficacy necessitates additional research.	[16,36,37]
Calcium (Ca^2+^)	78	62–95	Signal transduction	Ca acts as an intracellular signaling agent involved in proliferation, differentiation, apoptosis, ferroptosis, and enzyme regulation.	The mineral composition, including Ca, has been suggested as a potential factor in the observed biological effects of ZW.	The biological impacts are contingent upon calcium levels, cellular absorption, and physiological control. The impact of Ca found in ZW has not been independently verified.	[16,31,38,39]
Magnesium (Mg^2+^)	20	19–21	Enzyme cofactor	Mg is involved in enzymatic processes, ATP metabolism, DNA production, and cellular stress reactions.	Mg is one of the minerals identified in ZW and may enhance its overall mineral composition.	Normal or slightly elevated Mg concentrations may enhance immunotherapy efficacy and survival. The medicinal significance of Mg derived from ZW necessitates additional research.	[16,31,40,41,42]
Potassium (K^+^)				K modulates membrane potential, maintains intracellular ion equilibrium, and influences cellular function.	The ionic composition of ZW is influenced by the presence of K.	K is a vital electrolyte; it regulates cell proliferation and enhances anti-tumoral effects; nevertheless, there is no clear evidence supporting the anticancer effects of K levels achieved through ZW intake.	[43,44,45,46]
Selenium (Se)	0.0025	0.002–0.003	Antioxidant; regulates apoptosis; protects DNA	Se has a role in selenoproteins, antioxidant defense mechanisms, and the modulation of oxidative stress responses.	Se content has been identified as a possible factor in the antioxidant-related characteristics of ZW.	The biological effects of Se are highly dependent on dose. Se levels in ZW and their correlation with pharmacologically active Se exposure necessitate accurate quantitative analysis.	[46,47,48,49]
Zinc (Zn^2+^)	0.0137	0.0108–0.0166	DNA repair; antioxidant; immune defense	Zn plays roles in DNA repair, immune system modulation, antioxidant enzyme activity, and cellular signaling.	Zn has been identified as one of the trace minerals present in ZW.	Despite the known biological roles of Zn, it remains unclear how trace Zn in ZW contributes to anticancer activity.	[15,41,50,51]
Iron (Fe)	0.0808	0.0749–0.0867	Oxygen transport; chelation suppresses tumor proliferation	Fe is involved in oxygen transport, mitochondrial metabolism, and redox processes. Oxidative stress may also result from too much Fe.	The presence of Fe influences the total mineral makeup of ZW. The biological significance of Fe is contingent upon its chemical composition, concentration, and bioavailability.	The biological effects of Fe depend on its oxidation state, concentration, and cellular availability. The role of Fe from ZW requires quantitative assessment.	[15,47,52,53]
Lithium (Li^+^)	0.022	0.012–0.032	Modulates GSK-3β pathway; affects apoptosis	Li can influence intracellular signaling pathways, including pathways related to glycogen synthase kinase-3β (GSK-3β) regulation.	Li has been detected in some mineral waters, including reports describing ZW composition.	Biological activity of Li depends on dose and pharmacological exposure. Trace Li levels in ZW should not be equated with therapeutic Li administration.	[47,54,55]
Fluoride (F^−^)	1.5	—	Incorporated in anti-metabolite drugs; blocks DNA synthesis	F is a naturally occurring mineral ion involved mainly in dental mineralization and bone metabolism at appropriate exposure levels.	F has been reported as a component of ZW mineral composition.	The presence of F in ZW should not be interpreted as equivalent to fluorinated pharmaceutical compounds.	[31,47,56,57]
Arsenic (As)	0.0225	0.019–0.026	Pro-apoptotic; induces differentiation in cancer cells	As compounds can influence cellular stress pathways; however, As toxicity depends strongly on chemical form, dose, and exposure duration.	Some studies have reported trace levels of arsenic in ZW, whereas others have reported undetectable levels.	Trace As in water cannot be compared with As_2_O_3_, an approved pharmaceutical compound with a specific chemical form and controlled dosing. The safety and biological relevance of As in ZW require careful evaluation.	[16,46,58,59]
Strontium (Sr)	0.75	0.70–0.80	Bone remodeling; targets metastatic bone lesions	Sr may influence bone mineral metabolism and mitigate oxidative stress. It has been studied in particular medicinal preparations.	Sr may be present as a trace element in mineral waters.	The presence of microscopic amounts of Sr in ZW does not indicate pharmacological efficacy, as therapeutic outcomes depend on the compound’s formulation, dosage, and bioavailability.	[46,47,60,61]

The biological significance of ZW’s chemical composition arises from the synergistic and concentration-dependent actions of its ionic constituents. Calcium and magnesium serve as cofactors in over 300 enzymatic reactions, including those regulating DNA repair and apoptosis [62,63]. Selenium, an essential micronutrient with well-established anticancer and immunomodulatory roles, enhances glutathione peroxidase and thioredoxin reductase activity, thereby reducing oxidative DNA damage [22,64]. Lithium, at trace physiological concentrations, modulates the Wnt/β-catenin and GSK-3β pathways, influencing cell proliferation and neuroprotection [23,65]. Arsenic, while toxic at high levels, exerts selective pro-apoptotic effects at trace doses, as evidenced by the clinical success of arsenic trioxide in treating acute promyelocytic leukemia [24,26,66,67,68]. The coexistence of these ions in a single aqueous matrix could confer synergistic biochemical effects through redox regulation, pH modulation, and ionic signaling.

The alkaline nature of ZW (pH ≈ 8.0) may also contribute to its reported biological effects by influencing extracellular acid–base homeostasis. Tumor cells typically generate an acidic microenvironment due to elevated glycolytic activity and lactate efflux, conditions that favor invasion, metastasis, and therapeutic resistance. Correction of extracellular pH toward alkalinity has been shown to suppress cancer cell migration and improve drug sensitivity [27,28]. Thus, ZW’s intrinsic alkalinity could theoretically modulate the tumor microenvironment, rendering it less conducive to cancer progression.

Despite these promising physicochemical attributes, compositional variability across ZW samples collected at different times or depths has been reported [16], likely reflecting minor geological or seasonal fluctuations. Therefore, future research should prioritize establishing a standardized reference profile of ZW’s elemental and isotopic composition. Advanced analytical techniques, such as ICP–mass spectrometry (MS) (ICP-MS) and isotopic ratio MS, could provide a robust foundation for reproducibility and inter-laboratory comparison. Establishing such standardization will be essential for validating mechanistic findings and ensuring the translational reliability of biomedical research involving ZW.

## 3. Pharmacological Properties of ZW

The pharmacological profile of ZW encompasses a broad spectrum of biological activities, including antioxidant [69,70], anti-inflammatory [19], antidiabetic [19,46], antimicrobial [17], neuroprotective [71,72], and potential anticancer effects [73,74,75]. These properties are attributed to its distinctive mineral composition, alkaline pH, and stable ionic equilibrium, which together influence redox balance, enzymatic activity, and cellular homeostasis [31,76]. Although many studies remain preliminary, the cumulative findings indicate that ZW exerts both systemic and cell-specific effects that may contribute to its overall bioactivity.

### 3.1. Antioxidant, Hepatoprotective, and Renoprotective Effects

A limited but growing body of preclinical evidence suggests that ZW may modulate oxidative-stress-associated tissue injury, particularly in experimental models of hepatic and renal toxicity. In rodent models of carbon-tetrachloride (CCl_4_)- and paracetamol-induced hepatotoxicity, administration of ZW has been reported to partially normalize serum markers of liver injury, including alanine aminotransferase (ALT), aspartate aminotransferase (AST), and alkaline phosphatase (ALP), alongside increases in endogenous antioxidant enzymes, such as superoxide dismutase (SOD) and catalase (CAT) [70]. Histopathological analyses in these studies further revealed attenuation of hepatocellular degeneration, reduced lipid peroxidation, and diminished inflammatory cell infiltration. While these findings are consistent with a hepatoprotective profile, mechanistic attribution remains largely inferential, as pathway-specific interventions and causal validation are lacking [77].

Comparable protective effects have been described in experimental nephrotoxicity models induced by gentamicin, lead acetate, or CCl_4_, in which ZW intake was associated with reductions in serum creatinine and urea levels, preservation of glomerular structure, and mitigation of tubular injury [20,70]. These renal effects have been interpreted as reflecting enhanced antioxidant capacity and improved redox buffering, potentially involving mineral-dependent antioxidant enzymes such as glutathione peroxidase (GPx) and catalase. However, direct experimental evidence linking ZW mineral composition to enzyme activation or renal cytoprotection remains insufficient, and nonspecific factors, including hydration status, electrolyte balance, and osmotic effects, cannot be ruled out as contributing variables.

Supportive evidence for antioxidant and inflammation-modulating actions of ZW has also been reported in experimental wound-healing models [77]. In these studies, ZW administration was associated with accelerated wound closure and attenuation of local inflammatory responses, accompanied by reduced expression of pro-inflammatory cytokines, including tumor necrosis factor-alpha (TNF-α), interleukin-1 beta (IL-1β), and interleukin-6 (IL-6) [77]. These observations suggest that modulation of inflammatory signaling may contribute to the reported wound-healing activity; however, the evidence is primarily correlative and does not establish direct causal pathways. Independent experimental findings have similarly highlighted the importance of antioxidant defense and cytokine suppression in regulating tissue repair processes [78,79], lending conceptual support to these interpretations.

The biological effects observed in hepatic, renal, and wound-healing models have been attributed in varying ways to the physicochemical properties of ZW, including its alkaline pH, ionic composition, and trace-element content. Zinc, in particular, has been implicated in the regulation of inflammatory signaling, oxidative stress responses, and membrane stabilization, providing a plausible, though unconfirmed, mechanism for the observed anti-inflammatory profiles. In addition, experimental studies have suggested that certain trace elements, including arsenic, may modulate immune responses by affecting Th1- and Th2-associated cytokines and IL-1β signaling [77,78,79]. Importantly, arsenic is a recognized toxicant, and any immunomodulatory effects reported in experimental systems must be interpreted with caution and should not be construed as evidence of therapeutic benefit or safety.

Taken together, available data support a conceptual framework in which ZW may influence redox-sensitive and inflammatory pathways under conditions of experimentally induced tissue injury. However, the current evidence base is characterized by a small number of heterogeneous preclinical studies, limited mechanistic interrogation, and an absence of controlled clinical investigations. Consequently, definitive conclusions regarding the hepatoprotective, renoprotective, or wound-healing efficacy of ZW in humans cannot yet be drawn. Future research should prioritize standardized compositional analyses, rigorous dose–response assessments, mechanistic dissection of antioxidant and cytokine signaling pathways, and well-designed translational studies to determine whether these preclinical observations have clinical relevance.

### 3.2. Metabolic Modulation and Antidiabetic Activity

In addition to organ protection, ZW exhibits promising metabolic regulatory properties. Experimental studies have shown that ZW intake significantly reduces blood glucose and insulin levels in diabetic rodent models without causing hypoglycemia in normal controls [80]. The underlying mechanisms may involve improved insulin sensitivity, activation of AMP-activated protein kinase (AMPK), and enhanced oxidative enzyme function, possibly due to the lithium and magnesium content that influences carbohydrate metabolism [81,82]. Furthermore, the alkalinity of ZW may improve intracellular pH stability, indirectly enhancing enzymatic efficiency within glycolytic and mitochondrial pathways [83,84,85]. While these findings support a potential role for ZW in glucose homeostasis, no human clinical trials have yet validated its antidiabetic efficacy, and further mechanistic studies are warranted.

### 3.3. Neuroprotective and Neuromodulatory Actions

Trace lithium and magnesium in ZW are known modulators of neuronal excitability and neurotransmission. Preliminary studies have suggested that ZW consumption may exert mild anxiolytic and neuroprotective effects through lithium-mediated inhibition of glycogen synthase kinase-3β (GSK-3β), a key regulator of neuroplasticity and apoptosis [16,72]. These ions may also stabilize the neuronal membrane potential and modulate calcium signaling, thereby reducing excitotoxicity. Although these observations align with the known biological effects of lithium, controlled studies specifically assessing ZW’s neurological effects are lacking.

### 3.4. Antimicrobial and Immunomodulatory Properties

ZW exhibits moderate antibacterial activity against a variety of Gram-positive and Gram-negative pathogens, attributed to its mineral salinity, alkaline pH, and trace elements such as zinc and selenium [15]. This bacteriostatic property may explain ZW’s long-standing resistance to microbial contamination despite prolonged storage under ambient conditions. Moreover, several studies report immunomodulatory effects, including enhanced macrophage activity and upregulation of lymphocyte proliferation in animal models, suggesting a broader influence on immune homeostasis [31]. While these findings remain preliminary, they suggest that ZW could modulate immune function in ways relevant to cancer prevention and therapy.

## 4. Cytotoxic and Anticancer Effects of ZW

The potential anticancer activity of ZW has been explored across a variety of preclinical models, with several studies reporting cytotoxic, antiproliferative, and pro-apoptotic effects in diverse tumor types. The in vitro studies reviewed below have employed varying experimental conditions, including different ZW concentrations, pH adjustments, and treatment durations. A recurring limitation across several of these studies is the lack of appropriate control conditions to rule out nonspecific cell death due to factors such as pH fluctuations, osmotic imbalance, or nutrient deprivation. For example, when high concentrations of ZW (up to 50% *v*/*v*) are used, diluting the standard culture medium can itself induce cellular stress [14]. Similarly, the mildly alkaline pH of ZW (approximately 8.0) may, in the absence of pH-matched controls, confound the interpretation of biological outcomes. The following subsections critically evaluate the available evidence with these methodological considerations in mind, highlighting both the reported effects and their inherent limitations. A comparative summary of the experimental outcomes associated with ZW across different cancer models is provided in Table 2. It is important to understand that the mechanistic connections put forth in this section, which link particular mineral components of ZW (such as selenium, lithium, and arsenic) to the cellular effects seen in cancer cell lines that have been treated, are derived from parallel bodies of the literature on these elements separately rather than from dose-defined exposure studies utilizing ZW itself.

### 4.1. Antiproliferative and Apoptotic Activity in Lung Cancer

Lung cancer remains the foremost cause of cancer-related mortality worldwide, largely attributable to late-stage presentation, aggressive tumor biology, and the emergence of resistance to conventional and targeted therapies [86]. Despite notable advances in surgical techniques, cytotoxic chemotherapy, radiotherapy, molecularly targeted agents, and immune checkpoint inhibitors, long-term outcomes remain unsatisfactory, with five-year survival rates remaining approximately 20% [87]. This poor prognosis is driven by multiple factors, including intratumoral heterogeneity, adaptive resistance mechanisms such as secondary EGFR mutations, and limited efficacy of immunotherapy across diverse patient populations [88]. Consequently, there is a compelling need to identify novel therapeutic strategies that can effectively suppress tumor growth while minimizing systemic toxicity, particularly those capable of modulating key processes such as uncontrolled proliferation and evasion of programmed cell death.

**Table 2:** Proposed mechanisms and biological outcomes associated with Zamzam water across different in vitro cancer models.

Cancer Types	Proposed Contributing Factors	Proposed Mechanisms/Associated Effects	^#^ Biological Outcomes	Limitations	References
Lung Cancer	Arsenic, Cadmium, Alkaline pH, Arsenic Trioxide (As_2_O_3_)	Associated with cytotoxicity, G_0_/G_1_ cell-cycle arrest, and predominantly non-apoptotic (necrotic) cell death; putative apoptotic effects inferred from arsenic-associated mechanisms	Reduced A549 lung cancer cell viability with minimal cytotoxicity toward normal human skin fibroblasts	The biological relevance of these findings requires confirmation in additional lung cancer models and in vivo systems.	[75,89]
Colon Cancer	Sodium, Calcium, Magnesium, Potassium, Mitochondrial ROS	Associated with G_2_/M and S-phase arrest, mitochondrial dysfunction, ROS accumulation, apoptosis induction, and reduced proliferative capacity	Z1 treatment arrested ~38% of cells at the G_2_/M phase, whereas Z2 treatment caused ~42% of cells to accumulate in the S phase, accompanied by reduced cell viability	The contribution of individual minerals or unidentified components of ZW remains unclear. Quantitative relationships between mineral concentration, cellular exposure dose, and biological response have not been established.	[11,14,74,90]
Breast Cancer	Sodium, Calcium, Magnesium Phosphates	Associated with mitochondrial and cytoplasmic ROS accumulation, apoptosis induction, cell-cycle disruption, and modulation of MAPK-associated signaling pathways	Natural pH ZW produced a greater reduction in cell viability than pH-adjusted ZW. Early and late apoptotic populations were increased relative to untreated controls	Validation using multiple breast cancer subtypes (e.g., hormone receptor-positive and triple-negative models) and normal breast epithelial controls is required.	[89,91,92,93]
Hepatic Cancer	Selenium, Vanadium, Iron	Associated with mitochondrial dysfunction, oxidative stress, apoptosis induction, and reduced cellular proliferation	ZW increased apoptosis in HepG2 cells to approximately 38% and induced dose-dependent mitochondrial impairment with reduced cell viability	Further mechanistic investigations focusing on specific molecular targets are necessary to elucidate the mineral composition of ZW and its associated hepatocellular responses.	[94]
Glioblastoma	Sodium, Potassium, Calcium, Magnesium	Associated with modulation of cAMP signaling and µ-opioid-receptor-associated signaling pathways following chronic morphine exposure	Reduced cAMP overshoot following co-treatment with methadone and ZW, suggesting modulation of signaling pathways linked to opioid tolerance and dependence	Evidence remains preliminary, with limited mechanistic validation and the absence of comprehensive in vivo confirmation.	[95,96]

^#^ The reported correlations between certain trace elements or minerals and the cellular outcomes listed above are based on phenotypic observations in treated cells and compositional analysis of ZW; element-specific dose–response or concentration–effect data are not included. To date, no study has identified the amount or duration of ZW consumption required to replicate the in vitro exposure conditions used in these tests, nor has it isolated specific mineral ingredients from ZW to determine an active concentration. Therefore, rather than being established causal or quantitative links, the associations in Table 2 should be understood as compositional correlations.

In this regard, increasing attention has been directed toward naturally derived bioactive substances with potential anticancer properties. ZW, a naturally occurring alkaline mineral-rich water, has recently emerged as a candidate of interest due to its distinctive physicochemical composition and reported biological activity. Evidence from in vitro investigations using the A549 human lung carcinoma cell line indicates that ZW exerts measurable cytotoxic and antiproliferative effects. Specifically, two formulations, Z1 (pH-adjusted to 7.2) and Z2 (native alkaline form), were shown to significantly reduce cancer cell viability, demonstrating that its anticancer activity is not solely dependent on pH modification [75]. Notably, the native formulation (Z2) exhibited a more pronounced biological effect, characterized by induction of cell death predominantly through necrotic pathways rather than classical apoptosis. This effect was accompanied by arrest of the cell cycle at the G_0_/G_1_ phase, indicating disruption of cell-cycle progression and suppression of proliferative capacity. Importantly, neither cytoplasmic nor mitochondrial ROS levels were significantly altered following treatment, suggesting that the cytotoxic effects of ZW in lung cancer cells may proceed through ROS-independent mechanisms [75]. These findings distinguish ZW from many conventional anticancer agents that rely on oxidative stress to induce cell death. It is important to note, however, that the original study did not include pH-matched or osmolarity-controlled conditions. Therefore, the observed reduction in cell viability and G_0_/G_1_ arrest could be partially attributable to the alkalinity of the ZW preparation or to osmotic stress from medium dilution, rather than to a specific cytotoxic action of ZW’s mineral composition.

Morphological analyses revealed cellular shrinkage, nuclear condensation, and cytoplasmic vacuolization, all characteristic features of apoptotic cell death. Flow cytometric studies confirmed that ZW treatment promotes G_1_ and G_2_/M phase arrest, accompanied by reduced expression of cyclin D1, cyclin E, and cyclin B1 [66,67].

The underlying mechanism may involve the trace elements arsenic (As^3+^) and cadmium (Cd^2+^) present in ZW, which are known to initiate apoptosis through mitochondrial depolarization and activation of caspase-dependent pathways. Furthermore, the alkaline pH of ZW may perturb the acidic tumor microenvironment, impairing cellular metabolism and invasive potential [28].

Taken together, current evidence suggests that ZW exerts inhibitory effects on lung cancer cell growth through mechanisms involving cell-cycle arrest and non-apoptotic cell death pathways. Although preliminary, these findings highlight its potential as a complementary or exploratory agent in anticancer research and underscore the need for further mechanistic and translational studies to validate its therapeutic relevance.

### 4.2. Effects on Colon Cancer Cells

Colorectal cancer (CRC), encompassing malignancies of the colon and rectum, remains a major global health burden, ranking among the most frequently diagnosed cancers and a leading cause of cancer-related mortality worldwide [97]. Despite advances in screening and therapeutic interventions, CRC outcomes are often compromised by high recurrence rates, metastatic progression, and the development of resistance to chemotherapeutic agents [98]. These limitations highlight the urgent need for alternative or adjunctive strategies that can enhance treatment efficacy while reducing toxicity. In this context, increasing attention has been directed toward naturally derived compounds and dietary-based interventions, particularly those originating from bioactive food sources [97,98], due to their potential to modulate key pathways involved in tumor growth and progression.

ZW, a naturally alkaline water, has recently been investigated for its potential anticancer effects in CRC models. The anticancer potential of ZW has been extensively examined in human colon carcinoma (HCT-116) models, where it demonstrates strong antiproliferative effects. Comparative studies using two ZW preparations, Z1 (neutralized to pH 7.4) and Z2 (natural pH 8.0), showed that both formulations decreased cancer cell viability, with Z2 producing greater cytotoxicity (approximately 34% reduction in viability) [74]. Neither preparation exerted significant effects on normal fibroblasts, underscoring ZW’s apparent tumor selectivity.

Mechanistic assays revealed that ZW exposure leads to accumulation of mitochondrial reactive oxygen species (mtROS), mitochondrial membrane depolarization, and activation of intrinsic apoptotic cascades. Importantly, ROS production appeared to be localized to mitochondria rather than the cytosol, suggesting a targeted redox effect rather than global oxidative stress. Differential phase-specific arrest was also observed, with Z1-treated cells accumulating in G_2_/M and Z2-treated cells in S phase [74]. These data indicate that ZW triggers apoptosis through mitochondria-centered oxidative signaling and pH-dependent modulation of cell-cycle regulators.

In in vivo models, chronic administration of ZW (500 mL/day for 30 days) in rats led to a measurable reduction in colon tumor burden and improved histological parameters, including reduced nuclear pleomorphism and mitotic index [14]. While the Z1 and Z2 comparison in this study provides some insight into pH-dependent effects, it did not include inert, osmolarity-matched, or ion-depleted controls. Thus, the possibility remains that the observed cytotoxicity, particularly with the 50% *v*/*v* Z2 preparation, was influenced by non-specific factors such as osmotic shock and nutrient depletion rather than exclusively by ZW’s mineral composition. These findings support the translational potential of ZW as a complementary anticancer agent, although clinical corroboration remains absent.

Although experimental evidence remains limited, available studies collectively suggest that ZW disrupts CRC cell viability through coordinated interference with cell-cycle progression and redox homeostasis. Induction of G_2_/M arrest, together with elevated oxidative stress, is consistent with activation of mitochondria-dependent apoptotic pathways and may reflect the metabolic susceptibility of rapidly proliferating tumor cells. While these observations are currently restricted to in vitro models, they support further investigation into the mechanistic and translational relevance of ZW in CRC.

### 4.3. Antiproliferative Effects in Breast Cancer

Breast cancer remains the most frequently diagnosed malignancy worldwide, with approximately 2.3 million new cases and 685,000 deaths reported in 2020, and projections indicating a substantial rise in both incidence and mortality by 2040 [99,100]. This increasing burden reflects a multifactorial etiology, including environmental exposures, lifestyle-related risk factors—such as poor diet, physical inactivity, and alcohol consumption—and enhanced detection strategies. Among molecular subtypes, triple-negative breast cancer (TNBC) constitutes a particularly aggressive and heterogeneous group, defined by the absence of estrogen receptor, progesterone receptor, and HER2 expression [99]. TNBC disproportionately affects younger women and is associated with higher recurrence rates, limited therapeutic options, and inferior clinical outcomes relative to other breast cancer subtypes.

Against this backdrop, there is growing interest in identifying alternative agents that can modulate proliferative signaling and tumor cell survival. ZW has recently been explored in this context due to its distinctive mineral composition and physicochemical properties. The estrogen-receptor-positive MCF-7 cell line, one of the most extensively characterized in vitro models in breast cancer research, has served as a primary system for evaluating such effects [101,102,103,104,105,106]. In this model, ZW has been shown to induce oxidative-stress-mediated apoptosis and disrupt cell-cycle progression [89]. Specifically, exposure to ZW in its native alkaline form promotes mitochondrial depolarization, elevates intracellular ROS, and increases apoptotic cell fractions in a dose-dependent manner, with concomitant accumulation of cells in the G_2_/M phase [89].

Notably, divergent outcomes have been reported under different experimental conditions. In certain settings, ZW attenuated chemotherapy-induced apoptosis in breast cancer cells treated with agents such as cisplatin and doxorubicin. This effect was associated with activation of the MAPK pathway, including increased phosphorylation of CRAF, MEK1/2, and ERK1/2, alongside suppression of key apoptotic mediators such as caspase-3 and caspase-9 [91]. These contrasting findings suggest that the biological effects of ZW are highly context-dependent, influenced by factors such as cellular redox state, treatment combinations, and exposure duration. Such complexity underscores the need for systematic mechanistic studies to delineate its interactions with established therapeutic pathways before any consideration of clinical application. It is also noteworthy that the reported effects of ZW in breast cancer models are highly dependent on experimental conditions, including the pH of the ZW preparation and whether the water was co-administered with chemotherapeutic agents. However, none of the cited studies employed pH-matched, osmolarity-controlled, or ion-depleted control conditions, making it difficult to distinguish ZW-specific pharmacological effects from non-specific cellular responses to altered culture conditions.

### 4.4. Anticancer Effects in Hepatic and Glioblastoma Models

Beyond major epithelial malignancies, the biological activity of ZW has also been explored in hepatic and neural-derived tumor models, providing further insight into its mechanistic breadth. Although these cancer types differ substantially in their origins and therapeutic contexts, they offer complementary systems for examining the induction and modulation of apoptosis and intracellular signaling. Available evidence indicates that ZW exerts context-dependent effects, ranging from mitochondria-associated cytotoxicity in hepatic cancer cells to regulation of receptor-mediated signaling pathways in glioblastoma.

In human hepatocellular carcinoma (HepG2) cells, ZW demonstrates a pronounced pro-apoptotic effect, with apoptotic cell populations increasing from baseline (~0.84%) to approximately 38% following treatment with 50% ZW (*v*/*v*) [94]. However, it is crucial to interpret these findings with extreme caution, as the use of a 50% *v*/*v* concentration of ZW inherently introduces severe osmotic stress and nutrient deprivation, both of which are well-documented factors that trigger mitochondrial dysfunction and non-specific cell death. This response is accompanied by a dose-dependent reduction in cell viability and is mechanistically associated with mitochondrial dysfunction, consistent with activation of intrinsic apoptotic pathways. The mineral composition of ZW—which includes high concentrations of Na, Ca, and Mg, as well as trace elements such as Fe, Se, and V—is temporally linked to the observed anticancer activity; however, no study has isolated these components individually or determined their concentration-dependent influence on the effect. These components may contribute to the disruption of mitochondrial integrity and intracellular homeostasis, thereby promoting apoptotic signaling [94]. However, the precise molecular pathways underlying these effects remain to be fully defined.

In contrast, studies of glioblastoma multiforme (GBM), particularly those using the U-87 MG cell line, indicate that ZW primarily affects intracellular signaling rather than directly inducing cytotoxicity. In a morphine-conditioned model, co-treatment with ZW and methadone significantly attenuated the rebound increase in cyclic AMP (cAMP) levels following chronic morphine exposure [96]. Notably, the glioblastoma study did not report cytotoxicity as a primary outcome, and the observed modulation of cAMP signaling was not accompanied by pH-matched or ion-depleted controls. Whether the effects on signaling pathways reflect ZW-specific activity or non-specific ionic or osmotic influences remains to be determined. This finding suggests that ZW may modulate µ-opioid receptor-associated signaling pathways, potentially through its ionic composition. By suppressing cAMP upregulation, ZW appears to influence signaling processes linked to opioid tolerance and dependence, although its direct relevance to glioblastoma progression remains uncertain.

Collectively, these findings underscore the context-dependent nature of ZW’s biological activity, with pro-apoptotic effects predominating in hepatic cancer models and modulation of signaling observed in glioblastoma systems. Although the current evidence is largely preclinical, these observations expand the mechanistic framework of ZW and support further investigation into its potential roles across diverse cancer contexts.

### 4.5. Emerging Mechanistic Themes Across Cancer Models

Although the biological effects of ZW vary across tumor types, several recurring mechanistic patterns emerge from the currently available evidence. Across epithelial cancer models, including lung (A549), colorectal (HCT-116), and breast (MCF-7) cells, ZW consistently exhibits antiproliferative activity associated with disruption of cell-cycle progression and reduced cellular viability [14,75,89,107]. However, the phase of cell-cycle arrest and the mode of cell death appear to differ across tumor contexts, suggesting that ZW may interact with distinct regulatory pathways depending on cellular metabolic state and signaling background.

A second recurring feature is the involvement of mitochondrial dysfunction and oxidative stress responses. In colorectal, breast, and hepatocellular carcinoma models, ZW exposure has been associated with increased reactive oxygen species (ROS) generation, mitochondrial depolarization, and activation of apoptotic pathway cascades [74,89,94]. In contrast, lung cancer cells exhibit reduced viability in the absence of substantial ROS induction, indicating that ZW-mediated cytotoxicity may also proceed through ROS-independent mechanisms [75]. These observations collectively point toward mechanistic heterogeneity across cancer systems while supporting a broader role for mitochondrial and redox-associated processes.

ZW has also been reported to influence intracellular signaling pathways beyond direct cytotoxicity. In glioblastoma models, co-treatment with ZW and methadone modulated cyclic AMP (cAMP) dynamics following chronic morphine exposure [96], whereas breast cancer studies have implicated MAPK-related signaling in context-dependent modulation of apoptosis [91]. These findings suggest that the biological activity of ZW may extend beyond the induction of cell death to include broader effects on cellular signaling and stress adaptation.

The use of specific cancer cell lines has limited the available evidence, despite in vitro research offering initial insights into the biological effects of ZW in several cancer models. Every cancer cell line embodies a distinct molecular composition and fails to entirely capture the diversity of human malignancies. Consequently, results derived from models—such as MCF-7 breast cancer cells, HCT-116 colorectal cancer cells, or A549 lung cancer cells—should be interpreted as being specific to those experimental models rather than representative of all breast, colorectal, or lung cancers. Future studies should evaluate the effects of ZW across multiple cancer cell lines representing distinct molecular subtypes, together with appropriate non-tumorigenic counterpart cells, to determine tumor selectivity, reproducibility, and the underlying biological mechanisms. As an illustrative example, breast cancer investigations incorporating luminal (MCF-7) and triple-negative (MDA-MB-231 and MDA-MB-468) cell lines, together with non-tumorigenic mammary epithelial cells (MCF10A), would provide a more comprehensive assessment of the biological effects of ZW [108,109].

Collectively, these observations indicate that ZW may function as a context-dependent modulator of cancer cell behavior, influencing interconnected processes involving proliferation, mitochondrial integrity, oxidative balance, and intracellular signaling. The mechanistic basis of these effects, including potential contributions from mineral composition and alkaline pH, is explored in the following section. The integrated mechanistic patterns emerging across different cancer models are summarized in Figure 1.

In vivo confirmation of antitumor efficacy is significantly more limited than the corresponding in vitro literature across the cancer models reviewed in this section. Three investigations conducted thus far offer direct in vivo corroboration. Prolonged oral treatment with ZW (500 mL/day for 30 days) has been associated with reduced colon tumor load and improved histological features in a rat model [14]. ZW administration over a 12-day period significantly reduced the tumor-specific growth rate compared with tap-water controls in a separate study using a mouse model of mammary adenocarcinoma. This was accompanied by increased tissue glutathione, decreased malondialdehyde, and histopathological evidence of tumor necrosis [110]. The most recent study in a rat model of cadmium-induced brain tumorigenesis found that 30 days of ZW administration reduced Bmi-1 oncogene expression and increased p53 tumor suppressor expression in brain tissue. This is one of the few reports that links ZW exposure to a defined molecular tumor-suppressor/oncogene axis in vivo; however, ZW’s antioxidant effects (catalase, MDA, and glutathione) were not statistically significant, which is at odds with previous reports [111].

These three studies collectively represent the complete existing in vivo anticancer evidence for ZW. All studies were constrained by limited sample sizes, the lack of dose-ranging or survival analyses, and the absence of independent validation; two were published in journals with minimal editorial resources, and none used a validated xenograft or genetically engineered mouse model with randomization and blinding. No studies have evaluated prolonged survival outcomes or determined a dose–response correlation for tumor growth suppression in vivo. The organ-protective results outlined in this review (Section 6)—encompassing hepatoprotective, nephroprotective, and cardioprotective effects in chemically induced injury models—must not be mistaken for, nor interpreted as indirect evidence of, direct antitumor efficacy in vivo; safeguarding non-malignant tissue from toxic or drug-induced damage is mechanistically and functionally separate from inhibiting tumor proliferation.

### 4.6. Mechanistic Origins of the Context-Dependent ZW Effects

ZW induces apoptosis in some cancer models (e.g., human colon HCT-116 and liver HepG2 cells) but protects cancer cells in others (notably breast and ovarian lines treated with chemotherapy). For example, Al-Doghaither et al. [74] found that ZW (especially unbuffered at pH 8) sharply reduced HCT-116 viability and increased late apoptosis and ROS generation, and Abd-Rabou et al. [94] reported that 50% ZW killed 38% of HepG2 cells via apoptosis. In contrast, Siraj et al. [91] showed that in breast/ovarian cells, ZW increased proliferation and suppressed drug-induced apoptosis by upregulating ERK1/2–MAPK signaling. These opposing outcomes clearly go beyond a trivial “context-dependent” note and demand a deeper analysis. Possible explanations are as follows:

**Genetic/phenotypic differences between cell models:** Different cancers (and even different cell lines of the same cancer) have distinct mutations, receptor profiles, and signaling networks. ZW may engage disparate pathways in each context. For instance, colon and liver cells appeared to undergo ZW-induced oxidative stress and mitochondrial damage, whereas breast cancer cells showed strong ZW-induced activation of pro-survival ERK1/2 [89,91]. Lineage-specific factors (e.g., hormone receptors, oncogene status, p53 activity) could thus dictate whether ZW tips the balance toward death or survival. It is well known that even isogenic cell lines can diverge in drug response due to genetic drift, so intrinsic differences in these models may underlie the contradiction.

**pH-dependent drug neutralization:** ZW’s natural alkalinity (pH: 8) can alter the chemistry of weak acid/base drugs. In the colon study, unadjusted ZW (pH: 8) produced more apoptosis than pH-neutralized ZW, implying that higher pH amplified its effect (via increased ROS) [74]. Conversely, an alkaline extracellular pH can ionize certain chemotherapeutic agents or promote their degradation, reducing intracellular uptake [112]. For example, many weakly basic drugs become protonated (and less membrane-permeable) at high pH. If ZW raises the medium’s pH, it may partially “neutralize” drugs like doxorubicin or cyclophosphamide, attenuating their potency and allowing cancer cells to survive [91,113]. This could explain why, in the breast cancer experiments, ZW (pH-adjusted to 7.4 in that study) still blunted apoptosis—even slight pH shifts or buffering capacity of ZW may have been enough to interfere with the drugs’ action.

**Batch-to-batch variability of ZW:** Natural mineral waters vary in composition over time and source. ZW contains high levels of Na, Ca, Mg and trace elements (e.g., vanadium, iron, selenium), some with known biological effects [16]. Small fluctuations in ion concentrations or contaminants could dramatically change its activity. Siraj et al. used three different ZW samples (all buffered), but if one sample had higher levels of antioxidant minerals, it might better scavenge ROS, whereas another, richer in pro-oxidant metals, could induce cell death [91]. In other words, ZW is not a uniform reagent—batch differences in pH, mineral content, or microbial load could contribute to inconsistent outcomes.

**Treatment sequence and kinetics:** The order and timing of ZW versus chemotherapy exposure might matter. In the published breast cancer work, ZW and drug were co-administered for 24 h. If ZW precedes chemotherapy, it may prime cells by activating MAPK and anti-apoptotic pathways, whereas if chemotherapy is given first (or ZW added later), some apoptotic signaling might already be underway [91]. Temporal factors (e.g., pretreatment, simultaneous, or post-treatment) could thus alter the interaction. This is known for other drug combinations: a protective agent may only confer resistance if present at the right time. To resolve this paradox, systematic studies are needed.

Experimental recommendations:

- **Cross-model comparison:** Test ZW effects on a panel of cell lines (multiple breast, colon, liver, etc.) under identical conditions. Include both pH-adjusted and native ZW. Measure viability, apoptosis markers, ROS, and MAPK activation in each line. This will show whether certain lineages consistently respond in one direction or another.
- **Controlled pH/drug assays:** Repeat chemotherapy in media with and without ZW, carefully monitoring pH. Use analytical chemistry (e.g., HPLC) to check the stability/uptake of each drug in the presence of ZW. For example, compare doxorubicin retention in cells at pH 7.4 vs. pH 8.0. This will reveal if ZW’s alkalinity chemically neutralizes the drug.
- **Signal inhibition studies:** Use specific inhibitors or genetic knockdown to dissect pathways. For instance, blocking ERK1/2 (as in Siraj et al. [91]) reversed ZW’s protection; similar experiments in colon or liver cells could test whether ROS or MAPK are essential. Likewise, assessing p53 or mitochondrial pathway involvement (e.g., via caspase assays) could clarify how ZW triggers apoptosis in some cells.
- **Sequential treatment designs:** Test different schedules, including pre-treating cells with ZW before chemotherapy, co-treating, or adding ZW after the drug. Compare the endpoints in each scenario. If sequential timing changes the outcome, that would pinpoint kinetic factors.
- **Batch characterization:** Analyze multiple ZW lots by ICP-MS and pH measurement (as performed by Abd-Rabou et al. [94]). Correlate the mineral profile of each batch with its biological effect. One could even synthesize artificial ZW with defined compositions to identify which components drive each response.

## 5. Molecular Insights into the Anticancer Effects of ZW

Building upon the recurring phenotypic patterns observed across cancer models, including cytotoxicity, cell-cycle arrest, and apoptosis, it is biologically plausible that ZW activity is influenced by its unique ionic composition and mildly alkaline pH. However, a critical distinction must be made between observed biological effects and proven mechanisms. The majority of the original studies reviewed here employed descriptive endpoints (cell viability, ROS, apoptosis) rather than direct molecular interventions (gene knockdown, pharmacological inhibitors, or ion depletion experiments). Consequently, while the mineral profile of ZW provides a plausible biochemical foundation for its reported activity, the mechanistic pathways depicted below and summarized conceptually in Figure 2 remain largely hypothetical and require rigorous experimental validation. The following subsections integrate known biological functions of the relevant ions with the available experimental data, framing these as testable hypotheses rather than established mechanisms.

### 5.1. Synergistic Action of Bioactive Mineral Ions

The compositional complexity of ZW distinguishes it from other mineral waters and likely contributes to its diverse biological activities. Trace elements—such as arsenic (As^3+^), selenium (Se), lithium (Li^+^), magnesium (Mg^2+^), and calcium (Ca^2+^)—act as bioactive cofactors in redox and signaling pathways [94]. Many of these elements are known to participate in redox regulation, enzyme function, and intracellular signaling.

Rather than acting independently, these ions are likely to exert synergistic effects on cellular homeostasis. Calcium functions as a critical second messenger capable of triggering mitochondrial permeability transition and apoptosis, whereas magnesium supports DNA repair and enzymatic stability [38]. Selenium and iron are closely linked to redox regulation and antioxidant defense systems [21,114,115], while lithium is known to influence glycogen synthase kinase-3β (GSK-3β) and downstream proliferative signaling pathways [65,116]. Arsenic, at low concentrations, has been shown to induce apoptosis through mitochondrial and caspase-dependent mechanisms [66,67]. It is crucial to emphasize that these roles are inferred from the established biological functions of these individual ions and have not been directly validated within the specific context of ZW. The original studies cited in this review did not employ direct molecular techniques, such as ion-depletion experiments, pharmacological inhibitors, or gene knockdown, to confirm that the concentrations or combinations of these ions in ZW are sufficient to elicit these specific effects. Furthermore, the literature demonstrates that the clinically effective concentration of As_2_O_3_ is typically in the micromolar range (approximately 0.5–2 µM), whereas the As concentration in ZW is approximately 22.5 µg/L (0.27 µM), a substantial difference that raises important questions about whether these ions, individually, are present at pharmacologically meaningful levels. However, these roles are primarily inferred from established biological functions of these ions and have not been fully delineated within the specific context of ZW.

It remains speculative whether the ions present in ZW act independently, additively, or synergistically to influence cellular homeostasis. The hypothesis that their combined effects may collectively modulate cellular signaling, redox balance, and metabolic stability is plausible and merits investigation. However, this model, depicted conceptually in Figure 2, should be regarded as a testable hypothesis rather than an established mechanism. Systematic studies using reconstituted mineral mixtures, ion-selective chelation, and pathway-specific inhibitors are urgently needed to delineate the relative contributions of individual ions and their potential interactions.

These observations, collectively, suggest that ZW may influence interconnected signaling pathways involved in cell survival, stress adaptation, and apoptotic regulation. However, given the methodological limitations discussed above, including the absence of direct molecular validation and the confounding effects of pH and osmotic stress, this model remains conceptual. The relative contributions of specific ions, their potential synergy, and the role of alkaline pH have not been definitively established. Future studies employing pathway-specific inhibitors, genetic perturbations, and systematic ion reconstitution experiments are required to transform these hypotheses into validated mechanisms. The proposed interactions between mineral constituents, alkaline pH, and experimentally observed anticancer effects of ZW are summarized in Figure 2.

### 5.2. Modulation of Oxidative Stress and Redox Homeostasis

ZW appears to influence oxidative stress in a bidirectional and context-dependent manner. In several cancer models, exposure to ZW has been associated with increased intracellular and mitochondrial ROS, contributing to mitochondrial dysfunction and activation of intrinsic apoptotic pathways [74,89]. This effect is consistent with the concept of oxidative stress overload, whereby cancer cells, already operating near their maximal redox threshold, are selectively driven toward apoptosis by further ROS elevation [117].

Conversely, in non-malignant cells, ZW has been reported to support antioxidant defenses, including the activity of enzymes such as superoxide dismutase, catalase, and glutathione peroxidase [89]. This apparent dual behavior suggests that ZW may function as a redox modulator, amplifying oxidative stress in cancer cells while preserving redox balance in normal tissues. Such differential effects are consistent with established principles of redox biology in cancer [118], although the precise contribution of individual mineral components to these dynamics remains to be clarified.

### 5.3. Cell-Cycle Regulation and DNA Damage Response

Disruption of cell-cycle progression represents a consistent feature of ZW exposure across multiple cancer models. Reported effects include arrest at either the G_0_/G_1_ or G_2_/M phases, depending on cellular context and experimental conditions [74,75,89]. These alterations are associated with reduced expression or activity of key regulators, including cyclins and cyclin-dependent kinases, suggesting interference with checkpoint control mechanisms. Such effects are compatible with activation of DNA damage response pathways, including checkpoint kinase signaling, although direct mechanistic validation remains limited. Redox imbalance and ionic perturbations are well-recognized modulators of checkpoint activation and may underlie these observations. Overall, these findings indicate that ZW may constrain tumor growth not only by inducing cell death but also by restricting proliferative capacity through cell-cycle control.

Although oxidative stress modulation has been proposed as one of the potential biological effects associated with ZW, its direct impact on DNA repair mechanisms remains largely unexplored [80,119]. DNA damage responses involve coordinated pathways, including homologous recombination and non-homologous end joining, which maintain genomic stability and influence cancer cell survival. Given the reported antioxidant-related effects of ZW, it is possible that modulation of cellular redox status may indirectly influence DNA repair processes; however, this hypothesis has not yet been experimentally validated. Future studies should investigate whether ZW affects DNA damage sensing, activation of repair pathways, or the expression of key DNA repair regulators using molecular and functional assays.

### 5.4. pH-Mediated Modulation of the Tumor Microenvironment

The slightly alkaline pH of ZW (~7.8–8.0) introduces an additional dimension to its potential biological activity [74]. Tumor tissues are characterized by an acidic extracellular microenvironment, driven by aerobic glycolysis and lactate accumulation, which promotes invasion, immune evasion, and therapeutic resistance [27]. Alkalinization of this environment has been proposed as a strategy to counteract these effects and enhance treatment efficacy [28,120].

In this context, ZW may transiently influence extracellular pH, potentially disrupting proton-dependent transport systems such as the Na^+^/H^+^ exchanger and monocarboxylate transporters. Such perturbations could impair metabolic adaptation, reduce lactate efflux, and alter intracellular pH gradients. Although direct in vivo evidence remains limited, this hypothesis is consistent with current models of tumor microenvironment regulation and metabolic targeting [1].

### 5.5. Integration of Intracellular Signaling Pathways

Current evidence suggests that the biological activity of ZW cannot be attributed to a single linear mechanism but instead involves coordinated modulation of multiple intracellular signaling networks. Across different cancer models, ZW has been associated with alterations in pathways governing apoptosis, stress adaptation, proliferation, and metabolic regulation, indicating a highly context-dependent mode of action.

One of the most consistently implicated pathways involves mitochondrial apoptotic signaling. In several cancer models, including colorectal, breast, and hepatocellular carcinoma cells, ZW exposure has been associated with mitochondrial dysfunction, increased ROS generation, and activation of intrinsic apoptotic cascades [74,89,94]. These effects are consistent with mitochondrial membrane destabilization and subsequent activation of downstream caspase-mediated signaling. However, the relative contribution of ROS-dependent versus ROS-independent mechanisms appears to vary among tumor systems, suggesting that mitochondrial stress may represent a convergent but not universal mediator of ZW activity.

ZW has also been associated with modulation of MAPK signaling pathways. In breast cancer models, activation of the CRAF–MEK–ERK axis was reported following co-treatment with chemotherapeutic agents, coinciding with attenuation of apoptosis under certain experimental conditions [91]. These findings suggest that ZW may, in specific contexts, activate adaptive stress-response pathways that promote transient cytoprotective signaling rather than direct cytotoxicity. Such context-dependent effects underscore the complexity of ZW-mediated signaling interactions and highlight the importance of treatment conditions and cellular state in determining biological outcome.

In addition to MAPK signaling, ZW has been linked to modulation of cyclic-AMP (cAMP)-associated pathways in GBM models. Co-treatment with ZW and methadone reduced cAMP overshoot following chronic morphine exposure in U-87 MG cells, suggesting a potential influence of µ-opioid-receptor-associated signaling dynamics [96]. Although these effects were not directly associated with the induction of apoptosis, they indicate that ZW may influence receptor-mediated signaling and intracellular second-messenger systems independently of cytotoxic activity.

Beyond MAPK and cAMP signaling, ZW has also been shown to modulate the AMPK/mTOR pathway in a non-malignant renal injury model, where it enhanced autophagic flux and suppressed apoptotic signaling [18]. Given the established role of AMPK/mTOR signaling in cancer cell metabolism and autophagy-dependent survival, this finding suggests that similar pathway modulation may occur in malignant tissue; however, this has not been directly investigated in any cancer model, and extrapolation from a non-malignant renal system to tumor biology would be premature.

The biological effects associated with ZW may involve complex interactions among multiple intracellular signaling networks rather than activation of a single pathway. In particular, NRF2-mediated antioxidant responses and MAPK signaling pathways exhibit extensive crosstalk in regulating oxidative stress, inflammation, cell survival, and cellular adaptation [121]. Furthermore, certain metals and trace elements have been reported to influence aryl hydrocarbon receptor (AhR) and NRF2 signaling, which can interact with MAPK-dependent responses. Therefore, the diverse mineral composition of ZW may provide a biochemical basis for modulating interconnected stress-response pathways. However, direct evidence demonstrating that whole ZW or specific mineral components activate AhR, NRF2, or MAPK pathways is currently insufficient. Future studies using pathway-specific approaches, molecular profiling, and controlled evaluation of individual mineral contributions are required to determine the precise mechanisms underlying ZW-associated cellular responses.

## 6. Histological and Biochemical Effects of ZW

The histological and biochemical effects of ZW have been extensively evaluated in preclinical models, revealing consistent evidence of tissue protection, antioxidant activation, and metabolic restoration in multiple organ systems. These findings collectively suggest that ZW possesses cytoprotective properties that may extend beyond direct anticancer activity to broader systemic benefits, including hepatic, renal, cardiac, and reproductive protection. While the precise mechanisms underlying these effects remain to be fully elucidated, available data support the view that ZW mediates organ-level protection primarily through redox regulation, ionic stabilization, and anti-inflammatory modulation.

### 6.1. Hepatoprotective Mechanisms

Several studies have demonstrated that ZW confers substantial hepatoprotection against chemically induced liver injury. In animal models of carbon tetrachloride (CCl_4_) and paracetamol toxicity, ZW administration resulted in marked normalization of serum hepatic biomarkers, including alanine aminotransferase (ALT), aspartate aminotransferase (AST), and alkaline phosphatase (ALP), accompanied by increased levels of endogenous antioxidants, such as glutathione (GSH), superoxide dismutase (SOD), and catalase (CAT) [69,122]. Histological examination recognized near-complete restoration of hepatocyte morphology, reduction in necrosis and inflammatory infiltration, and preservation of sinusoidal architecture.

Mechanistically, these hepatoprotective effects appear to be mediated by activation of the Nrf2–ARE (nuclear factor erythroid 2–related factor 2–antioxidant response element) pathway, which upregulates cytoprotective genes and antioxidant enzymes [20,123]. Selenium and magnesium, abundant in ZW, likely act as cofactors in glutathione peroxidase and catalase catalysis, enhancing redox resilience and attenuating lipid peroxidation [46,124,125]. Furthermore, the mild alkalinity of ZW may neutralize metabolic acidosis associated with hepatotoxic stress, thereby stabilizing mitochondrial integrity and cellular energy balance [126]. These findings position ZW as a potential natural hepatoprotective agent, though controlled dose–response studies remain necessary to confirm its clinical relevance.

### 6.2. Renal Protection and Nephrohistological Integrity

ZW also exhibits notable nephroprotective effects in experimental models of renal injury induced by nephrotoxins such as gentamicin, lead acetate, and CCl_4_ [14,127]. Treatment with ZW was associated with decreased serum urea and creatinine levels, restoration of normal glomerular structure, and suppression of tubular necrosis and interstitial edema. These biochemical and morphological improvements correspond with enhanced activities of antioxidant enzymes and reduced accumulation of lipid peroxidation products.

At the molecular level, ZW may exert these effects through selenium- and magnesium-dependent activation of antioxidant defenses, combined with maintenance of osmotic and ionic homeostasis within renal tubular cells [31,76]. The presence of trace lithium may further contribute to renoprotection by modulating GSK-3β signaling, which plays a role in ischemia–reperfusion injury [128]. These data support the interpretation that ZW mitigates renal oxidative stress through coordinated ionic and enzymatic mechanisms, though validation in chronic exposure and human studies remains lacking.

A more recent, mechanistically detailed study examined ZW’s effect on cyclosporine A (CsA)-induced nephrotoxicity in rats, providing molecular insights beyond earlier biochemical and histological reports [18]. In this model, ZW co-administration significantly reversed CsA-induced renal apoptosis, evidenced by decreased expression of procaspase-8, caspase-8, caspase-9, cytochrome c, calpain, and caspase-3, together with increased Bcl-2 expression. In parallel, ZW enhanced autophagic flux, indicated by upregulation of Beclin-1, LC3-II, and Atg5 and downregulation of P62, an effect mechanistically linked to activation of the AMPK/mTOR/ULK1 pathway. These findings provide one of the more detailed molecular characterizations of ZW’s cytoprotective activity to date, implicating a specific, well-defined signaling axis (AMPK/mTOR) rather than only downstream phenotypic outcomes such as oxidative stress markers or histology.

### 6.3. Cardioprotective and Vascular Effects

Evidence also indicates that ZW may confer cardioprotective effects by modulating oxidative stress and ionic homeostasis. In models of isoproterenol-induced myocardial injury and high-fat-diet-induced cardiotoxicity, ZW consumption improved myocardial architecture, reduced inflammatory infiltration, and normalized cardiac biomarkers, including lactate dehydrogenase (LDH) and creatine kinase–MB (CK-MB) [76,129,130]. These effects were accompanied by increased antioxidant enzyme activity and decreased malondialdehyde (MDA) levels, reflecting attenuation of lipid peroxidation.

The cardioprotective mechanism is likely multifactorial. The balanced sodium–potassium ratio in ZW supports electrophysiological stability, while magnesium and calcium ions contribute to myocardial contractility and enzymatic homeostasis [41,131]. In addition, the alkalinity of ZW may counteract metabolic acidosis induced by ischemic or oxidative stress, improving cardiac energy metabolism [129,130]. Although these data highlight the therapeutic potential of ZW in cardiovascular contexts, no controlled clinical investigations have yet verified these outcomes in human populations.

### 6.4. Reproductive and Endocrine Implications

Limited evidence suggests that ZW may influence reproductive physiology, particularly in male rodents. Chronic administration of ZW has been associated with increased testicular weight, enhanced sperm count and motility, and elevated serum testosterone levels [20]. Histological analysis revealed improved spermatogenic organization and preserved seminiferous tubule structure compared with controls [20]. These findings may be explained by magnesium-mediated activation of steroidogenic enzymes and reduction in oxidative stress within the testes, contributing to improved endocrine function [46,132,133].

However, research in this domain remains preliminary. Data on female reproductive and hormonal effects are lacking, and potential endocrine-disruptive or long-term impacts have not been investigated. As such, these observations should be regarded as exploratory and require further verification through comprehensive studies of reproductive toxicity and endocrine function.

### 6.5. Histopathological Interpretation

Across organ systems, the histological and biochemical findings consistently demonstrate that ZW exerts systemic antioxidant and cytoprotective effects. The convergence of evidence from hepatic, renal, and cardiac models suggests that ZW’s unique ionic profile enhances endogenous defense mechanisms by upregulating antioxidant enzymes and stabilizing cellular ion gradients [69]. These properties may indirectly contribute to ZW’s anticancer potential by reducing systemic oxidative load and improving tissue resilience during carcinogenic stress or treatment-induced cytotoxic stress.

Nevertheless, despite promising preclinical outcomes, most studies are limited by small sample sizes, a lack of standardized histological scoring, and inconsistent dosing protocols. Few reports quantify the kinetics of biochemical recovery or employ molecular assays to validate mechanistic hypotheses. Furthermore, long-term safety data on ZW consumption at therapeutic volumes are absent, leaving the potential cumulative effects of trace metals—such as arsenic and lithium—uncertain.

## 7. Combination Therapy and Clinical Perspectives

### 7.1. Zamzam Water as a Potential Therapeutic Adjunct

Emerging preclinical evidence suggests that ZW may serve as a therapeutic adjunct, enhancing the efficacy and safety profiles of conventional cancer treatments. Its multifaceted biological properties, ranging from redox modulation and ionic homeostasis to microenvironmental pH regulation, provide a plausible foundation for combinatorial use with chemotherapeutic agents and natural bioactive compounds. In several in vitro and animal studies, ZW co-administration has been shown to mitigate drug-induced oxidative toxicity while maintaining or even augmenting cytotoxic efficacy against cancer cells [14,89].

Mechanistically, these synergistic effects may arise from the interplay between ZW’s antioxidant constituents and its alkaline buffering capacity. By stabilizing the redox balance and extracellular pH, ZW may counteract oxidative injury to normal tissues and reduce systemic inflammation, two major contributors to chemotherapy-related adverse effects [75,91,129]. For instance, in rodent models of cisplatin and doxorubicin toxicity, ZW supplementation reduced hepatic and renal oxidative markers and normalized transaminase and creatinine levels without impairing the drugs’ anticancer activity [18,19]. These findings suggest that ZW, when used alongside standard chemotherapeutic regimens, could improve tolerability and functional recovery following cytotoxic exposure, although clinical confirmation remains pending.

### 7.2. Interaction with Chemotherapeutic and Natural Compounds

ZW’s ionic composition and buffering potential may also influence the pharmacodynamics of chemotherapeutic agents. The modulation of extracellular and intracellular pH could theoretically affect drug ionization, transport, and cellular uptake, especially for weakly acidic or basic agents [14,43,47,112,134]. This is particularly relevant for drugs such as doxorubicin, methotrexate, and paclitaxel, whose efficacy and distribution are influenced by tumor acidity [113]. ZW’s alkalinity could enhance intracellular drug penetration by attenuating proton-pump-driven resistance mechanisms in the tumor microenvironment.

In parallel, combinations of ZW with natural bioactive molecules such as methadone have demonstrated additive antiproliferative effects in several in vitro studies [96,135]. The potential synergy may result from enhanced modulation of ROS, stabilization of mitochondrial potential, and improved bioavailability of phytochemicals under alkaline conditions [75,89]. However, the available studies vary widely in design and are often limited by a lack of pharmacokinetic assessment and standardized controls. Thus, before ZW can be considered a viable adjuvant, its interactions with cytotoxic and natural compounds must be systematically characterized using controlled pharmacological models that assess both efficacy and safety endpoints.

### 7.3. Supportive and Palliative Care Implications

Beyond its potential as an adjuvant, ZW may offer value as a supportive or palliative aid for patients undergoing intensive cancer treatment [75,91]. Hydration, electrolyte homeostasis, and oxidative balance are critical determinants of treatment tolerance and overall well-being in oncology [136]. The balanced mineral composition of ZW, combined with its gentle alkalinity, could help alleviate chemotherapy-induced gastrointestinal discomfort, fatigue, and oxidative stress. Moreover, its antioxidant and anti-inflammatory properties may support mucosal recovery and reduce oxidative injury in non-tumor tissues, thereby improving quality of life during prolonged therapy [20,46,69].

Nevertheless, it is essential to emphasize that current claims of palliative benefit are largely based on anecdotal observations or small-scale preclinical studies lacking clinical validation. No randomized controlled trials have been conducted to evaluate ZW’s role in symptom management, hydration, or biochemical recovery among cancer patients. Rigorous clinical studies incorporating objective endpoints, such as fatigue scores, biochemical stress markers, and electrolyte balance, are necessary to substantiate any supportive or palliative claims. Until such evidence is available, ZW’s use in palliative contexts should be viewed as experimental and limited to complementary frameworks under medical supervision.

### 7.4. Safety and Toxicological Considerations

The physicochemical composition of ZW generally meets global safety standards for drinking water, although certain trace components warrant careful monitoring. Arsenic concentrations in ZW slightly exceed the World Health Organization’s recommended limit of 10 µg/L [15], but existing toxicological assessments indicate that intermittent consumption at normal volumes remains safe. The lithium content of ZW, while low, could theoretically accumulate in chronic users, warranting caution among individuals receiving lithium-based pharmacotherapy or those with renal impairment [47,107,137].

No long-term studies have assessed the bioaccumulation potential or systemic distribution of ZW’s trace elements under therapeutic dosing conditions. Therefore, clinical protocols involving ZW should incorporate regular monitoring of renal function, serum electrolytes, and hepatic enzymes. Moreover, interactions with drugs that undergo ion-dependent absorption, such as tetracyclines, bisphosphonates, or tyrosine kinase inhibitors, should be considered in future pharmacovigilance studies. Establishing safety profiles in chronic consumption and combination-therapy contexts will be an essential prerequisite for any clinical recommendation.

### 7.5. Ethical and Cultural Dimensions

ZW holds deep religious and cultural significance, particularly within Islamic traditions, where it is revered for its purity and sanctity. While this historical context contributes to public interest, it also necessitates a careful distinction between spiritual reverence and scientific evaluation. Clinical research involving ZW must maintain ethical neutrality and adhere strictly to evidence-based frameworks. Investigators should ensure informed consent and cultural sensitivity while avoiding theological overtones in scientific interpretation. Responsible communication of research findings is critical to prevent the dissemination of unverified therapeutic claims or the exploitation of religious sentiments for commercial purposes.

## 8. Current Limitations and Future Directions

Despite encouraging preclinical observations, the evidence supporting the anticancer activity of ZW remains preliminary and is subject to important methodological limitations. Most available studies have been conducted in vitro using a limited number of representative cancer models, including lung (A549), colorectal (HCT-116), breast (MCF-7), hepatocellular (HepG2), and glioblastoma (U-87 MG) cell lines, and have relied primarily on descriptive endpoints, such as cell viability, ROS generation, apoptosis, and cell-cycle distribution [19,74,75,89,91,94,96]. Although these studies provide an initial framework for mechanistic interpretation, they do not establish causal relationships between specific physicochemical features of ZW and the observed biological responses. The principal limitations of the current literature, along with a proposed roadmap for future mechanistic and translational investigations of ZW, are summarized in Figure 3.

A major unresolved question is whether the reported effects are attributable to the unique composition of ZW itself or reflect more general consequences of mineral supplementation and modest alkalinity. Few studies have incorporated appropriate comparator systems, such as synthetic mineral mixtures, pH-matched controls, or ion-depletion approaches, making it difficult to distinguish ZW-specific effects from those attributable to individual constituents or to nonspecific changes in ionic strength and osmolarity. In addition, potential variability in mineral and trace element composition has not been systematically addressed, which may affect reproducibility across studies and complicate standardization for translational development [16]. Another major unresolved pharmacological limitation is the significant discrepancy between the sub-micromolar/nanomolar concentrations of trace elements in ZW and the micromolar concentrations typically required for single-agent anticancer activity. Unless rigorous evidence for supra-additive synergy is provided, the contribution of these specific ions to the observed effects remains speculative.

The current evidence base also provides limited mechanistic resolution. In most studies, pathway involvement is inferred from phenotypic observations rather than established through direct functional interrogation. Consequently, the relative contributions of redox perturbation, mitochondrial dysfunction, cell-cycle regulation, and intracellular signaling remain incompletely defined. This limitation is particularly relevant in light of the context-dependent nature of ZW responses: while several studies reported pro-apoptotic and antiproliferative effects, Siraj et al. found that ZW attenuated chemotherapy-induced apoptosis across breast, colorectal, and ovarian cancer cell lines treated with paclitaxel, 5-fluorouracil, and cisplatin, respectively—an effect mediated by activation of the MAPK pathway and confirmed by reversal of this protection upon ERK1/2 silencing [89,91]. A key methodological distinction likely explains this divergence: in that study, ZW samples were buffered with PBS and adjusted to physiological pH (7.4) before treatment, neutralizing ZW’s native mild alkalinity (pH 7.9–8.0), whereas the pro-apoptotic effects reported elsewhere were observed using ZW in its native alkaline state [89]. Since alkalinity itself has been proposed as a contributor to ZW’s tumor-suppressive activity by correcting extracellular pH, testing a pH-neutralized preparation represents a fundamentally different exposure condition. Differences in cancer cell line panels and chemotherapeutic agents may also contribute. These findings underscore the importance of defining the molecular and physicochemical determinants, particularly pH state, that govern divergent biological responses to ZW.

Addressing these uncertainties will require a more rigorous and mechanistically integrated research strategy. Comprehensive physicochemical characterization, including mineral, trace element, isotopic, and pH profiling, should be coupled with systems-level approaches—such as transcriptomics, proteomics, metabolomics, ionomics, and redox profiling—to identify molecular networks and biomarkers associated with response [1,138,139]. Functional validation using pharmacological inhibitors, genetic perturbation, and synthetic reconstruction of ZW mineral composition will be essential for establishing causality and dissecting the contributions of individual ions and their combinatorial interactions. Advanced experimental models, including patient-derived organoids and tumor-bearing animal models, will further clarify whether the in vitro effects of ZW translate into meaningful antitumor activity in vivo [140].

The effects of long-term ZW consumption in combination with lifestyle-related factors, including tobacco smoking and alcohol consumption, remain largely unexplored. These factors may independently influence oxidative stress, inflammatory responses, and xenobiotic metabolism, thereby potentially modifying the biological effects of ZW. Furthermore, although ZW contains multiple minerals and trace elements with potential biological relevance (Table 1), current evidence is insufficient to determine whether ZW influences drug-metabolizing enzymes or transport systems [47]. In particular, the effects of ZW on cytochrome P450 enzymes, ATP-binding cassette transporters, solute carrier transporters, and the pharmacokinetic profiles of co-administered medications have not been systematically investigated. Future studies should evaluate these potential interactions using appropriate in vitro, in vivo, and clinical pharmacokinetic approaches to establish the safety profile and translational relevance of ZW consumption.

From a translational perspective, clinical investigation should proceed cautiously and only after robust preclinical standardization. Early-phase studies should focus initially on safety, tolerability, and supportive-care outcomes rather than assumptions of direct anticancer efficacy. Equally important will be the development of regulatory and ethical frameworks to ensure that any discussion of ZW in oncology remains evidence-based and avoids unsupported therapeutic claims (Figure 3).

Taken together, the available data support the view that ZW is a biologically intriguing physicochemical system, but they remain insufficient to define its therapeutic relevance. Establishing whether the unique mineral composition and mildly alkaline pH of ZW confer reproducible and clinically meaningful effects will require standardized physicochemical characterization, mechanistic dissection, advanced preclinical validation, and carefully designed clinical studies. Addressing these priorities will be essential not only to clarify the biomedical significance of ZW, but also to advance a broader understanding of how ionic composition, redox balance, and extracellular pH influence disease biology.

## 9. Conclusions

ZW is a naturally occurring water with unique chemical properties and biological activity that goes beyond its historic and cultural importance. Empirical research indicates that ZW exhibits antioxidant, anti-inflammatory, and cytoprotective properties in both in vitro and in vivo settings, although its direct anticancer benefits are primarily supported by in vitro evidence and a few limited in vivo studies. Organ-protective results should not be interpreted as proof of antitumor effectiveness. In cancer systems, the existing evidence suggests a context-specific mechanism of action encompassing oxidative stress, mitochondrial stability, cell-cycle progression, and intracellular signaling, culminating in the novel concept of ZW as an ionic-redox modulator, in which its mineral composition and slight alkalinity jointly affect cellular equilibrium.

Currently, the evidence is predominantly preclinical, and the molecular factors that influence ZW’s efficacy have not been defined with mechanistic precision. The specific contributions of different mineral components, their cooperative interactions, and the impact of alkaline pH are not fully elucidated, nor is the effect of the method of administration or delivery approach. The lack of uniform physicochemical characterization, suitable comparator systems, and regulated clinical trials prevents any determination of treatment effectiveness in humans.

Consequently, ZW ought not to be considered a clinically proven anticancer treatment. It embodies a biologically fascinating physicochemical system that provides a valuable framework for examining how ionic composition, redox equilibrium, and extracellular pH govern cellular activity. Establishing the translational significance of ZW, whether in cancer prevention, supportive care, or integrative oncology, will require systematic mechanistic research and meticulously designed clinical trials. In the absence of such proof, any evaluation of ZW in biomedical practice must adhere to scientific rigor, clinical prudence, and acknowledgment of its cultural and religious importance.

## Figures and Tables

**Figure 1:**
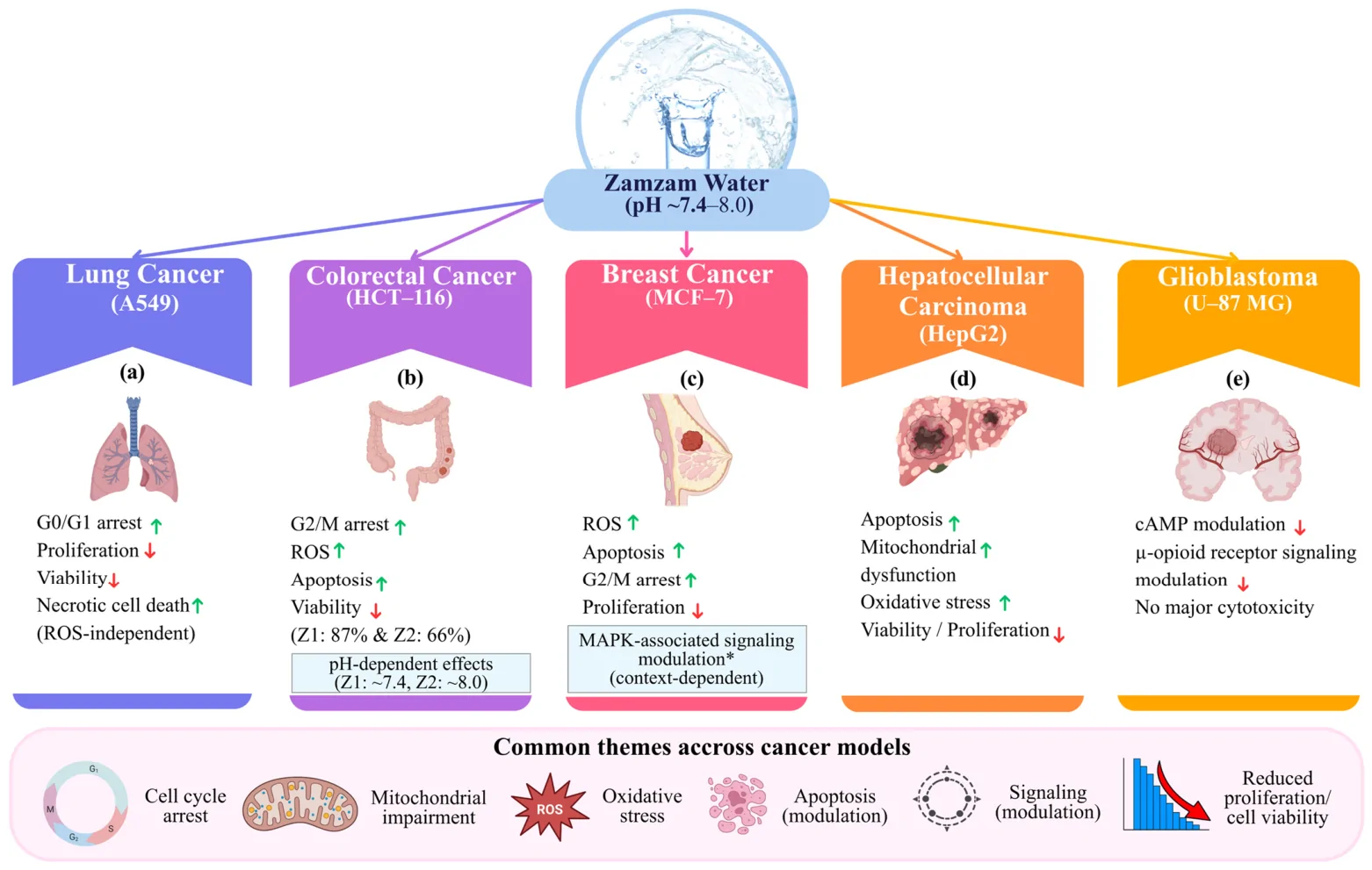
Putative mechanisms underlying the anticancer activity of Zamzam water across cancer models. (**a**) In A549 lung cancer cells, ZW induces G_0_/G_1_ arrest and non-apoptotic (necrotic) cytotoxicity, resulting in reduced viability. (**b**) In HCT–116 colorectal cancer cells, ZW promotes G_2_/M arrest, increases ROS, and induces apoptosis in a pH-dependent manner. (**c**) In MCF–7 breast cancer cells, ZW induces ROS, apoptosis, and G_2_/M arrest, and modulates MAPK-associated signaling in certain contexts. (**d**) In HepG2 hepatocellular carcinoma cells, ZW induces apoptosis, mitochondrial dysfunction, and oxidative stress, thereby reducing cell viability. (**e**) In U–87 MG glioblastoma cells, ZW modulates cAMP and µ-opioid-receptor-associated signaling without major cytotoxicity. Collectively, these findings suggest that ZW exerts context-dependent biological effects, modulating proliferation, mitochondrial integrity, oxidative balance, and intracellular signaling pathways across different cancer models. The cell and organ icons in this figure were obtained from BioRender.com [accessed on 10–20 January 2026]. * Observed in certain contexts/combination therapies; not universal across all settings. Green arrow: Increase/Up-regulation; Red Arrow: Decrease/reduce/down-regulation. Abbreviations: cAMP, cyclic AMP; MAPK, mitogen-activated protein kinase; ROS, reactive oxygen species; ZW, Zamzam water.

**Figure 2:**
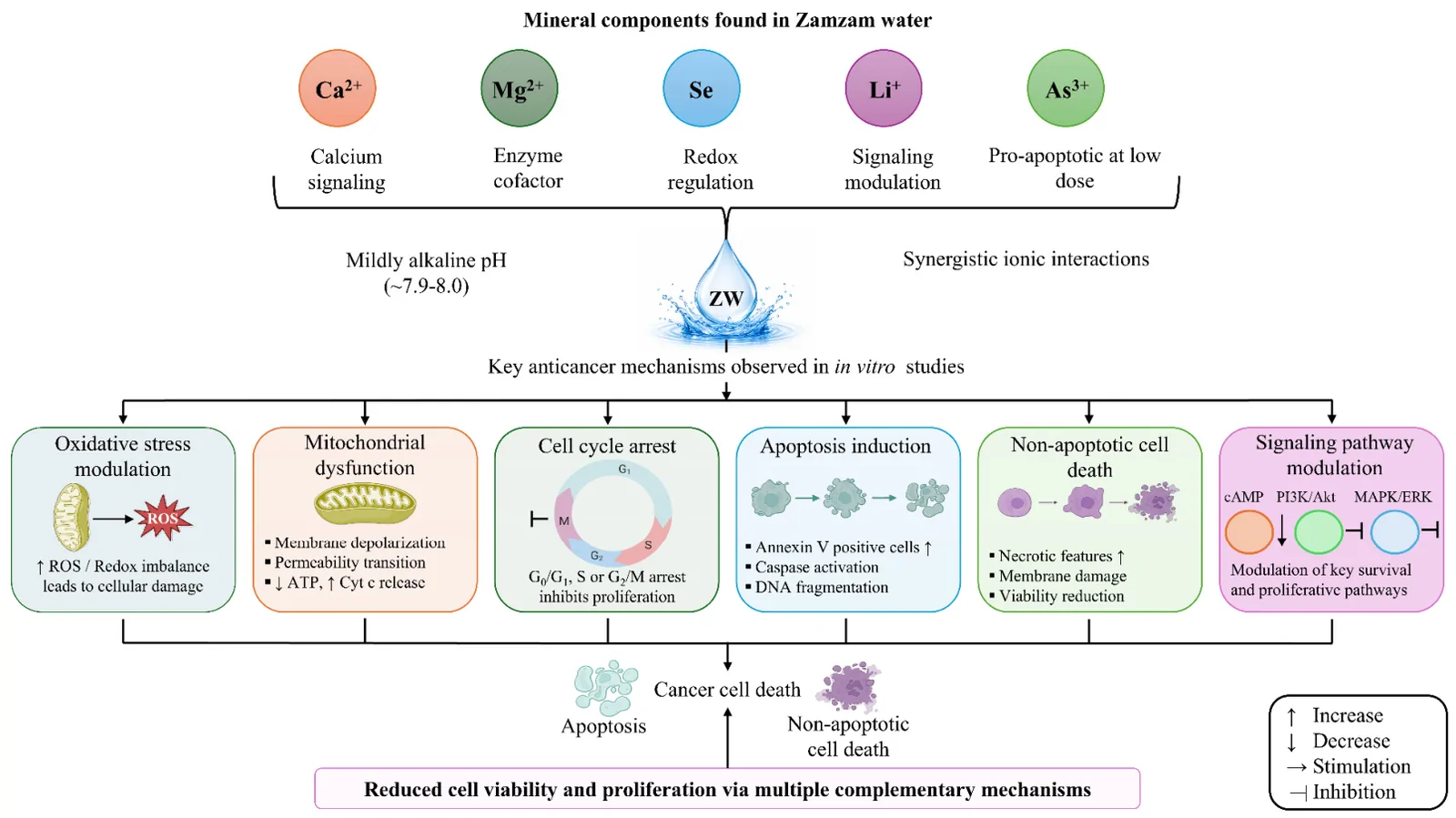
Conceptual working model of proposed mechanisms underlying the anticancer effects of Zamzam water. The distinctive mineral composition and mildly alkaline pH of ZW may collectively contribute to its reported biological effects across experimental cancer models. Major mineral constituents—including Ca, Mg, Zn, Se, and Li, as well as trace As-associated species—have been implicated in pathways associated with oxidative balance, mitochondrial function, and intracellular signaling. Experimental studies suggest that ZW exposure is associated with reduced cellular proliferation, induction of apoptosis or non-apoptotic cell death, and alterations in cancer cell morphology. These effects are proposed to involve modulation of redox homeostasis, mitochondrial integrity, and cell-cycle regulation. The figure summarizes the putative mechanisms inferred from currently available preclinical evidence. The cell and organ icons in this figure were obtained from BioRender.com [accessed on 10–20 January 2026].

**Figure 3:**
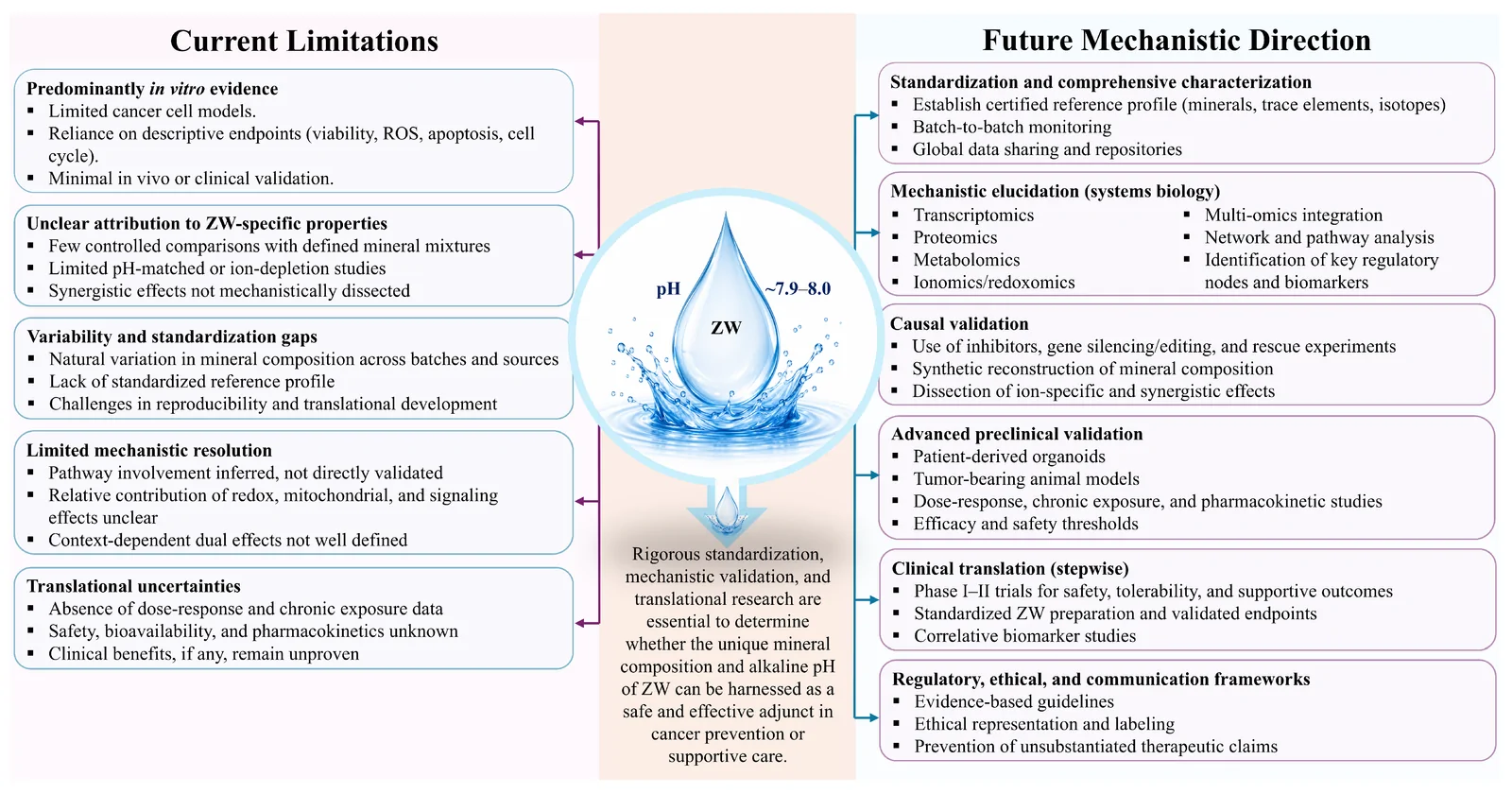
Current limitations and future research priorities for the mechanistic and translational evaluation of Zamzam water.

## Data Availability

No new data were created or analyzed in this study. Data sharing is not applicable to this article.
